# Comparative performance evaluation of FilmArray RP 2.1 and targeted next-generation sequencing in upper respiratory tract infections

**DOI:** 10.3389/fcimb.2025.1610445

**Published:** 2025-07-24

**Authors:** Wenxiang Jin, Zehan Dai, Pengyuan Zhu, Oliver Ma, Jiahao Li, Ava Kong, Junbin Wu, Feifei Liu, Miaozhi Li, Zixin Du, Dan Xue, Ganqiang Yang, Nafen Ye, Chunleung Lo, Chaohui Hu, Lei Zhang

**Affiliations:** ^1^ Department of Laboratory Diagnosis, KingMed Diagnostics, Guangzhou, Guangdong, China; ^2^ Research and Development Department, Guangzhou KingCreate Biotechnology Co., Ltd, Guangzhou, China; ^3^ KingMed Diagnostics, Hong Kong, Hong Kong SAR, China

**Keywords:** targeted next-generation sequencing, FilmArray RP 2.1 panel, upper respiratory tract infections, pathogen identification, multiplex PCR

## Abstract

**Introduction:**

Upper respiratory tract infections (URTIs) represent a significant global health burden, impacting patient morbidity and quality of life. The implementation of accurate pathogen detection methods is crucial for precise diagnosis and effective clinical management of URTIs. This study evaluates the clinical performance of targeted next-generation sequencing (tNGS) as a diagnostic tool for comprehensive identification of URTI-associated pathogens.

**Methods:**

A total of 190 nasopharyngeal swab specimens from patients were enrolled for the study. These specimens underwent pathogen identification using both tNGS and the FilmArray respiratory panel. The results obtained from these two methods were then compared.

**Results:**

Overall, tNGS identified 164 positive samples, compared to 91 positives identified by FilmArray. Regarding the shared target species or subtypes, tNGS identified 97 positive samples, whereas the FilmArray respiratory panel detected 88 positives out of 190 specimens. tNGS identified a diverse array of 34 different pathogens, significantly surpassing the 12 pathogens identified by the FilmArray panel. The detection rates for tNGS and FilmArray were 51.05% (97/190) and 46.32% (88/190), respectively. Statistical analysis revealed no significant difference in the detection rates of 10 specific respiratory pathogens (with ≥3 positives). Furthermore, the overall pathogen detection accuracy of tNGS was determined to be 90.16% (95%_CI_ = 83.45%~94.81%), with a sensitivity of 95.45% (88.77%~98.75%).

**Conclusions:**

The tNGS method demonstrates broader pathogen detection capability compared to the FilmArray, achieving a higher positive detection rate in upper respiratory tract infections. It demonstrates high accuracy and sensitivity, offering a viable and rapid diagnostic approach for upper respiratory tract infections.

## Introduction

Upper respiratory tract infections (URTIs) are acute infections caused by pathogenic microorganisms that affect the nose, nasal sinuses, pharynx, and larynx. These infections typically present with symptoms such as fever, headache, and coughing, and in rare cases, may progress to severe complications (Wang et al., 2021). Such complications can impose significant social and economic burdens on both individuals and society. Therefore, rapid and accurate diagnosis of upper respiratory tract pathogens is essential for the effective and rational use of anti-infective drugs. This not only reduces the risk of developing drug resistance but also expedites patient recovery (Liu et al., 2022).

Currently, various methods are available for the detection of respiratory pathogens, including culture, multiplex polymerase chain reaction (multi-PCR), metagenomic next-generation sequencing (mNGS), and targeted next-generation sequencing (tNGS) (Li et al., 2022; Lin et al., 2023; Cui et al., 2024). Conventional microbiological diagnostic methods for respiratory infections, such as microscopy, culture, and serology, are associated with several limitations. Microscopy often lacks sufficient sensitivity to detect low-abundance pathogens and cannot provide definitive identification. Culture-based methods, while specific, are time-consuming, typically requiring days to yield results, which delays timely diagnosis and treatment. Serology, which detects antibodies or antigens, may not reflect the current infection status due to the lag between pathogen exposure and antibody production, and it is less effective for detecting acute infections. These drawbacks collectively contribute to delayed diagnosis, inappropriate antibiotic use, and suboptimal patient outcomes in the management of respiratory infections.

Multi-PCR holds significant advantages in pathogen detection, including high sensitivity and specificity, rapid turnaround time, cost-effectiveness, and multiplexing capability. These features enable early and accurate diagnosis, therapeutic monitoring, and effective infection control. However, as the number of targets increases, the complexity of primer design for PCR-based assays escalates exponentially, which significantly restricts the expansion of target numbers in such diagnostic products. One such representative of PCR-based assays is the FilmArray RP2.1 EUA Panel (referred as FA RP 2.1). FA RP 2, produced by BioFire^®^ Diagnostics (Utah, United States), is an FDA-cleared multiplex PCR system (510(k) Number K223591) designed for the rapid detection of respiratory pathogens (<2h). It also permits immediate testing upon sample receipt, accommodating even a single sample without the need for batch processing. FA RP 2.1 demonstrates broad-spectrum coverage of clinically relevant respiratory pathogens through simultaneous detection of 18 viruses and 4 bacteria (**Supplementary Table S1**) (Kaku et al., 2018; Ferrer et al., 2023). FA RP 2. has been implemented in some hospitals globally and its diagnostic value has been demonstrated [Berry et al., 2022; Cassidy et al., 2022; Chang et al., 2022; Tazi et al., 2022], supporting rapid decision-making in respiratory infection management.

mNGS offers extensive pathogen coverage and a high positive detection rate, but the cost per assay is high. Besides, since individual DNA and RNA processing procedures cannot be combined directly, adapting the RNA procedure would further increase the cost of mNGS. tNGS involves multiplex PCR amplification on samples, followed by sequencing the amplified products using NGS. The high throughput feature of NGS makes it able to identify up to hundreds of target loci in one assay. tNGS panels are usually designed for specific infection scenarios, covering a limited spectrum, making them more streamlined and cost-effective. This method detects infection-related microbes with lower costs and allows for the simultaneous detection of multiple pathogens. Some reports have demonstrated the clinical application of tNGS (Flurin et al., 2022; Hong et al., 2023; Lin et al., 2023; Fourgeaud et al., 2024) in different types of infections. The deployment of tNGS for pathogen identification and its diagnostic value have been demonstrated in previous studies (Flurin et al., 2022; Hong et al., 2023; Lin et al., 2023; Fourgeaud et al., 2024). The initial findings suggest that all three methods could assist in clinical decision-making (Flurin et al., 2022; Hong et al., 2023; Lin et al., 2023; Fourgeaud et al., 2024). However, whether the broad-spectrum and cost-effective tNGS offers advantages over the FDA-approved FA RP 2 in detecting upper respiratory tract infections remains to be further investigated.

This paper aims to investigate the detection efficacy of tNGS technology for upper respiratory tract infections in comparison with FA RP 2.1. The findings of this study could have significant implications for the clinical and economic aspects of managing respiratory infections, potentially paving the way for more efficient and cost-effective diagnostic approaches.

## Materials and methods

### Study overview

**Figure 1:**
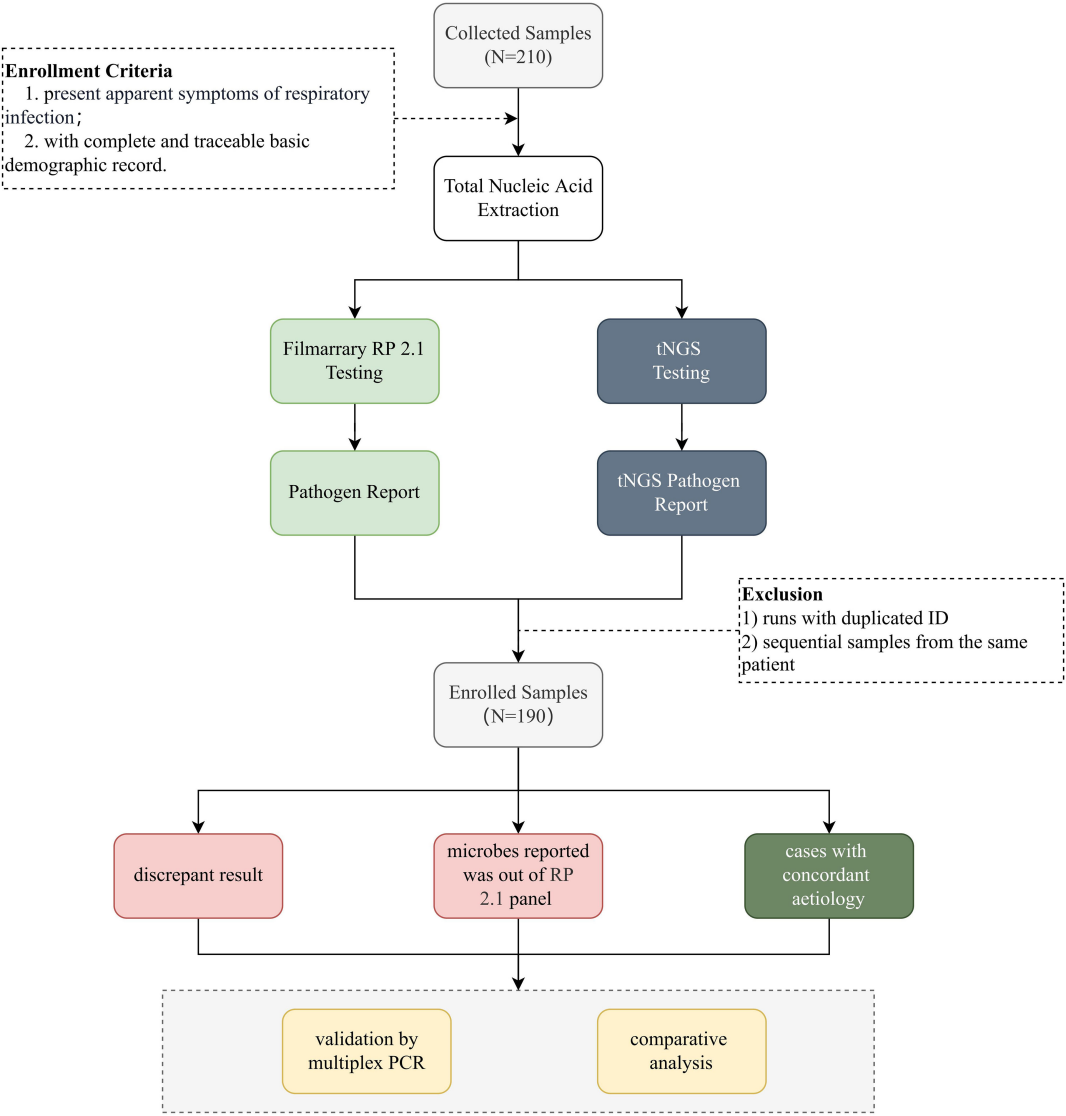
Schematic overview of the study. This figure provides a schematic representation of the study design. Nasopharyngeal swab samples were collected from individuals suspected of respiratory tract infections. After nucleic acid extraction, pathogens were identified using FA RP 2.1 and UP50 tNGS panel (Kingcreate, Guangzhou, China). A total of 190 deduplicated samples meeting predefined inclusion criteria were enrolled and subjected to downstream analysis. Discrepant results for specific pathogens between tNGS and FA RP 2.1 were further validated using qPCR.

This study is a retrospective analysis evaluating tNGS’s performance in detecting RTI pathogens in comparison with FA RP 2.1 (**Figure 1**). This study was conducted from October 2022 to January 2023, nasopharyngeal swab (NPS) specimens were collected from patients with suspected respiratory tract infections in KingMed Diagnostics to conduct laboratory FA RP 2.1 and tNGS testings. The specimens were immediately stored in disposable virus sampling tube at 4°C.

Total nucleic acid extraction was conducted within 24h after sample collection, and reverse transcription was immediately carried out thereafter. The cDNA and DNA mixture were stored at -80°C and tested within 7 days. Eligibility criteria: 1) all patients presented apparent symptoms of URTIs; 2) with complete basic information and demographic records. Samples with insufficient sample volume, samples with tNGS run failure, and sample collected from the same patients were excluded. tNGS was performed concurrently with the FA RP 2.1 assay. The reports generated by each method were interpreted independently without knowledge of the results from the other method. A comparative analysis was conducted to assess the performance of tNGS in comparison with FA RP 2.1. In cases of discordant results, additional qPCR testing was performed to evaluate the robustness of tNGS, provided that sufficient sample material remained available.

### Sampling and total nucleic acid extraction

Swab samples were collected and added to 3 mL of saline or transport medium. A volume of 1.3 mL from the swab sample was aliquoted, to which 13 μL of an exogenous internal reference (*Rhizobium trifolii*, 3.5×10^6 CFU/mL) was added. The mixture was subjected to vortexing for homogeneous mixing followed by centrifugation at 12,000 rpm for 5 minutes. The supernatant was carefully discarded, the pellet was resuspended in 400 μL (for manual extraction procedures) or 250 μL (for automated extraction systems) by pipetting up and down to ensure thorough mixing. The total nucleic acid was extracted from the clinical samples in 500 uL volume using MetaPure DNA & RNA Extraction Kit (KingCreate Co. Ltd., Guangzhou, China) in reference to the official instruction guide. The generated nucleic acid was resolved in DNase and RNase Free Water and stored at -80°C.

### FilmArray procedure

Nasopharyngeal swab samples were tested using the BioFire RP2.1 system (BioMérieux) according to the manufacturer’ s instructions. Specifically, 300 μL of each sample was mixed with 3 mL of viral transport medium and loaded into the BioFire pouches, which contain all necessary reagents for nucleic acid extraction, amplification, and detection in 45 minutes. For quality control, the BioFire pouch includes two internal controls. The software automatically interprets the endpoint melting curve data to provide a qualitative result for each target, with a microorganism reported as detected if at least one of its corresponding assays is positive. If either internal control fails, the software automatically provides a result of “Invalid” for all panel analytes.

### Targeted next-generation sequencing panel

#### Panel establishment

The panel was designed through systematic analysis of multiple sources, including clinical expert consensus documents and infection-related literature (Kasper and Fauci, 2016; Huang et al., 2020; Li et al., 2021; Liu et al., 2023). The resulting tNGS panel, encompasses 95 clinically relevant pathogen targets (**Supplementary Table S2**). The reference sequence data were primarily sourced from NCBI RefSeq/NT and were refined by removing highly similar redundant sequences. Target selection prioritized genes validated by established PCR methods, followed by bioinformatic evaluation of conserved and specific regions.

Primer design followed stringent criteria: (1) GC content maintained at 40-60%; (2) length optimized to 18–26 bp; (3) Tm values set at ~60°C; and (4) elimination of self-dimers, hairpins, and cross-dimers. The pathogen-specific multiplex PCR primers were designed by KingCreate Biotech (Guangzhou, China) and synthesized by Sangon Biotech (Shanghai, China). The accompanying PCR protocol was rigorously optimized to ensure high-sensitivity target amplification.

#### Library construction and sequencing

Library preparation was then performed using the Respiratory Pathogen Microorganisms Multiplex Testing Kit (KS608-50SHXD96, KingCreate, Guangzhou, China) following manufacturer’s guide. cDNA was synthesised by reverse transcription of the extracted nucleic acid, followed by steps such as target region enrichment, first round of purification, junction ligation and second round of purification to complete library construction. Nuclease-free water (Invitrogen, Waltham, MA, USA) was used as a non-template control (NTC) to detect contamination. Generated libraries were quantified using Equalbit DNA HS Assay Kit (Vazyme Biotech, Nanjing, Jiangsu, China) on an Invitrogen Qubit 3.0/4.0 Fluorometer (Thermo Fisher Scientific, Waltham, MA, USA). This ensured that all samples had a library density of at least 0.5 ng/μL, otherwise, the library would be subjected to re-construction. The constructed libraries were pooled to homogeneous mass. Qualified pooled library was diluted and denatured, of which 500 μL was loaded onto the KM Miniseq Dx-CN Sequencer (KY301, Kingcreate, Guangzhou, China) using a 2 x 150 bp paired-end sequencing protocol, as per the manufacturer’s instructions. Each batch’s external positive (*Bacilus subtilis*) and negative (saline) controls were run in parallel with the clinical specimens throughout the workflow, including nucleic acid extraction, library construction and sequencing, to detect contamination.

### Bioinformatics

The output sequencing data was transferred to fastq format using the bcl2fastq (https://github.com/brwnj/bcl2fastq). Subsequently, FastQC (https://www.bioinformatics.babraham.ac.uk/projects/fastqc/) and MultiQC (Ewels et al., 2016) were utilized for assessing overall sequencing quality. The fastp v0.20.1 (Chen et al., 2018) was then employed for adapter trimming and low-quality read filtration. The read filtration criteria are delineated as follows: 1) reads with an average quality score below 15 are subjected to trimming; 2) reads possessing the ambiguous “N” bases over 10; 3) reads with a length of < 15 bp.

The selected human reference genome (version hg19) and pathogenic target genome sequences were downloaded from the NCBI GenBank FTP site (https://ftp.ncbi.nlm.nih.gov/genomes/genbank/). The initial step involved aligning the reads from each sample with the human genome (GCF_000001405.13) using the BWA-mem (Li and Durbin, 2009), which served to filter out host sequences. Subsequently, the remaining amplified reads were aligned to the pathogen genomes utilizing the Bowtie2 v2.4.1 in a ‘very-sensitive’ mode (Langmead and Salzberg, 2012). Following this, the alignment depth and coverage were calculated with the assistance of Samtools (Li et al., 2009) and Bamdst v1.0.5 (https://github.com/shiquan/bamdst). The process specifically enumerated reads that aligned to the target amplicon regions with overlaps greater than 40 bp. By aggregating the counts of reads for each target amplicon, the resultant figures served as indicators of the pathogen presence corresponding to the target amplicons.

To call positive signals for specific pathogens, mapped reads were counted and normalized to read per 100,000 reads (RPhK). Cases with specific RPhK were considered positive. If a specific species or higher-level taxonomy unit was identified in a sample with RPhK value ≥ 10, this species/unit was regarded as “present” in this sample, or else reported as “absent”.

### Statistical analysis

The positive rate was calculated by dividing the number of positive samples by the total number of samples. The sensitivity and accuracy were calculated as defined in previous studies (Altman and Bland, 1994; van, Stralen et al., 2009). The comparison of detection rates was performed using chi-square test or *Spearman’s* rank correlation test, and a P value less than 0.05 was considered statistically significant. These statistics were computed using base R 4.3.1 (https://www.r-project.org/).

### Validation of tNGS results

In instances where discrepancies arose between tNGS and FA RP 2.1 results, PCR validation was conducted using the Resp40 PCR Kit (KS655-Resp40-48, Kingcreate, Guangzhou, China) on samples with sufficient remaining material. This kit is designed for the qualitative detection of nucleic acids from 45 common respiratory pathogens in human nasopharyngeal swabs, sputum, and bronchoalveolar lavage fluid samples. The comprehensive list of pathogen targets, including viruses, bacteria, fungi, and atypical pathogens, is detailed in **Supplementary Table S3**. This validation step aims to verify the additional detection made by tNGS relative to FA RP 2.1, thereby assessing the robustness and accuracy of tNGS.

## Results

A total of 190 samples were enrolled in this study following the inclusion and exclusion criteria, with redundant samples being excluded. Combined, tNGS and FA RP 2.1 detected a total of 35 pathogens. It is noteworthy that SARS-CoV-2 was the only pathogen detected exclusively by FA RP 2.1, as it was not within the target spectrum of tNGS.

Owing to its broader range of targets, tNGS was able to detect a greater number of pathogens across the entire study population, with pathogen-positive results reported in 164 cases (86.32%). In contrast, FA RP 2.1, which targets predominantly viral pathogens, reported pathogen-positive results in only 91 cases (47.89%) (**Table 1**).

**Table 1:** Detection frequencies for shared targets between FA RP 2.1 and tNGS.

Pathogen	tNGS	Filmarray	χ2	P-value
Human rhinovirus	48 (25.26%)	43 (22.63%)	3.200	0.074
Human adenovirus	13 (6.84%)	10 (5.26%)	1.333	0.248
Human respiratory syncytial virus	11 (5.79%)	11 (5.79%)	0.000	1.000
Influenza B virus	11 (5.79%)	6 (3.16%)	3.200	0.074
Influenza A virus	5 (2.63%)	6 (3.16%)	0.000	1.000
Human rubulavirus 2	6 (3.16%)	4 (2.11%)	0.500	0.480
Human metapneumovirus	5 (2.63%)	5 (2.63%)	NA	NA#
Human respirovirus 3	5 (2.63%)	4 (2.11%)	0.000	1.000
Human respirovirus 1	3 (1.58%)	4 (2.11%)	0.000	1.000
Human coronavirus OC43	3 (1.58%)	0 (0.00%)	1.333	0.248
Human orthorubulavirus 4*	2 (1.05%)	2 (1.05%)	NA	NA#
Human coronavirus HKU1*	2 (1.05%)	0 (0.00%)	0.500	0.480
Human coronavirus 229E*	1 (0.53%)	0 (0.00%)	0.000	1.000
Human coronavirus NL63*	1 (0.53%)	1 (0.53%)	NA	NA#
Bordetella pertussis*	1 (0.53%)	0 (0.00%)	0.000	1.000
Mycoplasma pneumoniae*	1 (0.53%)	0 (0.00%)	0.000	1.000
Shared species and subtype	97 (51.05%)	88 (46.32%)	7.111	0.008
Overall	164 (86.32%)	91 (47.89%)	73	< 0.001

*These pathogens were with total positves less than 3. # Not Available: all postives were concordant between two methods.

### Overall performance of tNGS in reference to FA RP 2.1

Given that FA RP 2.1 is a product with FDA clearance and has been clinically validated over multiple years demonstrating its performance and accuracy, we used it as a reference standard to evaluate the pathogen detection performance of tNGS. Owing to the lack of complete concordance in the pathogen spectrum, particularly given that the subtyping targets of one method may not be included in the other, we categorized our statistical analysis into two methods: one based solely on the shared subtyping targets and the other encompassing both species and subtypes. Taking Human rhinovirus as an example, tNGS can discriminate at the subtype level (e.g., Human rhinovirus A, Human rhinovirus B), whereas FA RP 2.1 only reports at a broader species level (Human rhinovirus). Due to this disparity in classification granularity, direct comparison was infeasible. Thus, for analytical consistency, all subtypes were merged and evaluated at the species level.

**Figure 2:**
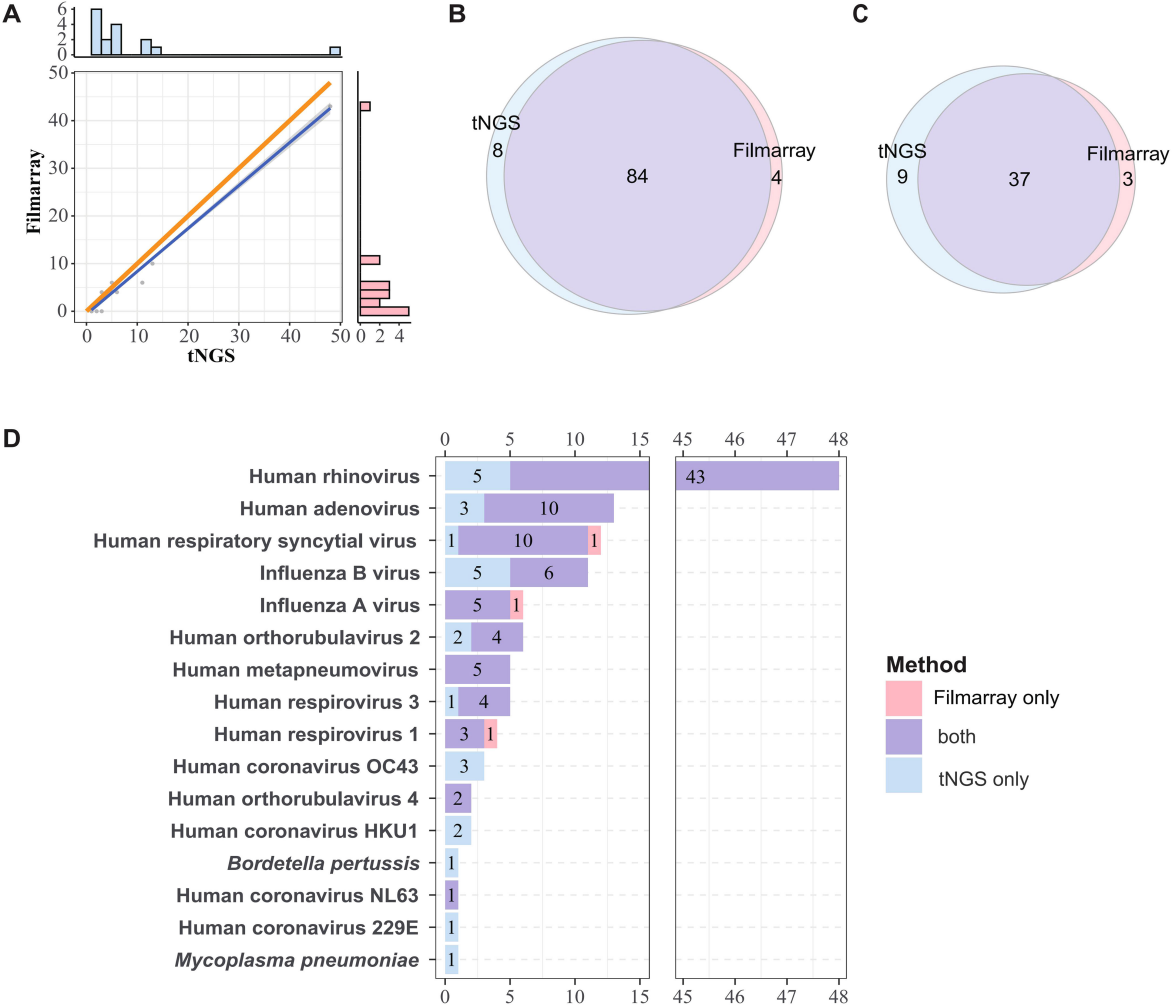
Comparative analysis of pathogen detection frequencies and concordance between methods. **(A)** Linear correlation of detection frequencies of tNGS and FA RP 2.1. The dots represent detection frequencies of specific microogranisms. The blue line represents the fitted line, and the gray area indicates the 95% confidence interval. The bar plots in the right and top show the marginal distributions of FA RP 2.1 and tNGS. The orange line serves as a reference line, representing the line of equality, which is the ideal fit line for the detection frequency when the two methods are in complete agreement. Correlation was determined using *Spearman* rank test (P < 0.01). **(B, C)** Overall concordance in detection frequencies at species and subtype Levels. **(D)** Overlap of detection frequencies of the shared targets between FA RP 2.1 and tNGS.

When considering species-level detection, tNGS demonstrated an accuracy of 90.16% (95%CI = 83.45%, 94.81%), sensitivity of 95.45% (88.77%, 98.75%), positive predictive value (PPV) of 91.30% (83.58%, 96.17%), and negative predictive value (NPV) of 86.67% (69.28%, 96.24%). At the subtype level, the accuracy was 84.00% (73.72%, 91.45%), PPV was 80.43% (66.09%, 90.64%), and NPV was 89.66% (72.65%, 97.81%) (**Table 2**). In brief, these stats demonstrated that tNGS carries high detection agreement with FA RP 2.1. This is further supported by the high correlation between the detection frequencies between both methods. As depicted in **Figure 2A**, there is a strong linear relationship between the detection frequencies of different pathogens by the two methods (*Spearman* test, P < 0.01). Most of the positives of tNGS were consistent with FA RP 2.1 (84/92 vs 84/88), as demonstrated in **Figure 2B**. Regarding the only targets at subtype level, most of the positives of tNGS were consistent with FA RP 2.1 (37/46 vs 37/40, **Figure 2C**).

**Table 2:** Overall performance of tNGS in reference to Filmarray.

Performance metrics	Species & subtype	Subtype
Accuracy	90.16%	84.00%
	(83.45%, 94.81%)	(73.72%, 91.45%)
Sensitivity	95.45%	92.50%
	(88.77%, 98.75%)	(79.61%, 98.43%)
Specificity	76.47%	74.29%
	(58.83%, 89.25%)	(56.74%, 87.51%)
Positive Predictive Value	91.30%	80.43%
	(83.58%, 96.17%)	(66.09%, 90.64%)
Negative Predictive Value	86.67%	89.66%
	(69.28%, 96.24%)	(72.65%, 97.81%)

Two statistical approaches were employed due to discrepancies in the targeted pathogens between the two methods. Specifically, one method may include subtyping targets not covered by the other. For instance, tNGS can identify Human rhinovirus at the subtype level (e.g., Human rhinovirus A, Human rhinovirus B), while FilmArray reports only at the species level (Human rhinovirus). This difference in classification granularity precluded direct comparison. Therefore, we conducted analyses in two ways: one considering only the shared subtyping targets and the other including both species and subtypes. For consistency, all subtypes were aggregated and assessed at the species level.

However, due to the higher number of additional pathogens detected by tNGS, both analyses revealed relatively lower specificity, 76.47% (58.83%, 89.25%) and 74.29% (56.74%, 87.51%), respectively (**Table 2**). All detailed performance metrics for all pathogens are summarized in **Supplementary Table S4**.

### Concordance of pathogen detection between FA RP 2.1 and tNGS

The consistency of pathogen detection between FA RP 2.1 and tNGS is summarized in **Figure 2D** (for shared targets only). Human rhinovirus was the most frequently detected pathogen (48 cases), followed by Human adenovirus (13 cases) and Human respiratory syncytial virus (12 cases), among others. For pathogens detected in more than three cases, FA RP 2.1 and tNGS exhibited a relatively high degree of concordance. For example, of the 48 Human rhinovirus-positive samples, 43 were positive by both methods; for Human adenovirus, the concordance was 10 out of 13 cases; and for Human respiratory syncytial virus, it was 10 out of 12 cases. However, for influenza B, only 6 out of 11 cases were positive by both methods, with the remaining five cases detected exclusively by tNGS. There was only one case each where FA RP 2.1 was positive and tNGS was negative for human respiratory syncytial virus, influenza A, and Human respirovirus 1. Pathogens/strains detected solely by tNGS included Human coronavirus OC43, Human coronavirus HKU1, Human coronavirus 229E, *Mycoplasma pneumoniae*, and *Bordetella pertussis*. Based on these information, the two methods demonstrated a certain degree of concordance in pathogen detection, with tNGS indicating higher sensitivity, as evidenced by the numerous cases positive by tNGS but negative by FA.

The detection landscape for all targets, inclusive of those not shared between the two methods, is depicted in **Supplementary Figure S1**. The following pathogens, not targeted by FA RP 2.1, were detected by tNGS: among Gram-positive bacteria, *Staphylococcus aureus* was detected in 41 cases and *Streptococcus* spp. in 28 cases. Among Gram-negative bacteria, *Klebsiella pneumoniae* was identified in 34 cases, *Moraxella catarrhalis* in 30 cases, *Acinetobacter baumannii* in 24 cases, *Stenotrophomonas maltophilia* in 22 cases, *Haemophilus influenzae* in 9 cases, and *Pseudomonas aeruginosa* in 8 cases. Additionally, Human bocavirus 1 was detected in 15 cases and Human gammaherpesvirus 4 in 14 cases. The broader detection spectrum gives tNGS the ability to report more pathogen types, while the FA RP 2.1 spectrum mainly covers viruses. tNGS yielded also bacteria and chlamydia/mycoplasma (**Supplementary Figures S2A, B**). This indicates that tNGS could serve as a good complement for FA RP 2.1.

In total, tNGS identified 34 distinct types of pathogens, significantly surpassing the 12 types detected by FA RP 2.1 (**Supplementary Figure S2C**). Notably, SARS-CoV-2 was uniquely detected by FA RP 2.1, as it fell outside the scope of tNGS targets. Among the pathogens detected by tNGS, 23 were not identified by FA RP 2.1., of which the majority are unique to tNGS and not shared with FA RP 2.1. However it is noteworthy that five pathogens detected only by tNGS - Human coronavirus OC43, Human coronavirus HKU1, Human coronavirus 229E, *M. pneumoniae*, and *Bordetella pertussis* - were within the detection target spectrum of FA RP 2.1. This underscores the enhanced detection capability of tNGS, even within the realm of shared targets.

### Verification of additional detections by tNGS

To validate the discordant results between FA RP 2.1 and tNGS, a multiplex qPCR detection method, specifically the commercial RP40 kit, was introduced to verify the differential detection outcomes for each pathogen. Given that the study was conducted some time ago, not all samples remained in sufficient quantity or were of adequate quality to validate the pathogens detected discordantly by FA RP 2.1 and tNGS. Finally, we managed to select 49 qualified samples to verify the certain detection results of tNGS, with the detailed information summarized in **Supplementary Figure S3** and **Table 3**.

**Table 3:** Verification of additional detections by tNGS.

SampleID	Pathogen	tNGS	Filmarray	PCR	Cross validation
Sample083	*Acinetobacter baumannii*	+	NA	–	tNGS_only
Sample060	*Acinetobacter baumannii*	+	NA	–	tNGS_only
Sample092	*Acinetobacter baumannii*	+	NA	–	tNGS_only
Sample028	*Acinetobacter baumannii*	+	NA	–	tNGS_only
Sample055	*Acinetobacter baumannii*	+	NA	–	tNGS_only
Sample082	*Acinetobacter baumannii*	+	NA	–	tNGS_only
Sample131	*Acinetobacter baumannii*	+	NA	+	PCR_validated
Sample021	*Acinetobacter baumannii*	+	NA	+	PCR_validated
Sample210	*Haemophilus influenzae*	+	NA	+	PCR_validated
Sample043	*Haemophilus influenzae*	+	NA	+	PCR_validated
Sample055	Human bocavirus	+	NA	–	tNGS_only
Sample019	Human bocavirus	+	NA	+	PCR_validated
Sample082	Human bocavirus	+	NA	+	PCR_validated
Sample086	*Klebsiella pneumoniae*	+	NA	–	tNGS_only
Sample060	*Klebsiella pneumoniae*	+	NA	–	tNGS_only
Sample098	*Klebsiella pneumoniae*	+	NA	–	tNGS_only
Sample065	*Klebsiella pneumoniae*	+	NA	–	tNGS_only
Sample055	*Klebsiella pneumoniae*	+	NA	–	tNGS_only
Sample056	*Klebsiella pneumoniae*	+	NA	+	PCR_validated
Sample144	*Klebsiella pneumoniae*	+	NA	+	PCR_validated
Sample122	*Klebsiella pneumoniae*	+	NA	+	PCR_validated
Sample038	*Klebsiella pneumoniae*	+	NA	+	PCR_validated
Sample114	*Klebsiella pneumoniae*	+	NA	+	PCR_validated
Sample026	*Klebsiella pneumoniae*	+	NA	+	PCR_validated
Sample063	*Moraxella catarrhalis*	+	NA	+	PCR_validated
Sample201	*Moraxella catarrhalis*	+	NA	+	PCR_validated
Sample094	*Moraxella catarrhalis*	+	NA	+	PCR_validated
Sample019	*Moraxella catarrhalis*	+	NA	+	PCR_validated
Sample079	*Moraxella catarrhalis*	+	NA	+	PCR_validated
Sample002	*Moraxella catarrhalis*	+	NA	+	PCR_validated
Sample083	*Pseudomonas aeruginosa*	+	NA	–	tNGS_only
Sample098	*Pseudomonas aeruginosa*	+	NA	–	tNGS_only
Sample210	*Pseudomonas aeruginosa*	+	NA	+	PCR_validated
Sample190	*Pseudomonas aeruginosa*	+	NA	+	PCR_validated
Sample167	*Pseudomonas aeruginosa*	+	NA	+	PCR_validated
Sample046	*Pseudomonas aeruginosa*	+	NA	+	PCR_validated
Sample186	*Staphylococcus aureus*	+	NA	–	tNGS_only
Sample145	*Staphylococcus aureus*	+	NA	–	tNGS_only
Sample115	*Staphylococcus aureus*	+	NA	–	tNGS_only
Sample098	*Staphylococcus aureus*	+	NA	–	tNGS_only
Sample065	*Staphylococcus aureus*	+	NA	–	tNGS_only
Sample026	*Staphylococcus aureus*	+	NA	–	tNGS_only
Sample137	*Staphylococcus aureus*	+	NA	+	PCR_validated
Sample110	*Staphylococcus aureus*	+	NA	+	PCR_validated
Sample131	*Staphylococcus aureus*	+	NA	+	PCR_validated
Sample092	*Staphylococcus aureus*	+	NA	+	PCR_validated
Sample122	*Staphylococcus aureus*	+	NA	+	PCR_validated
Sample207	*Staphylococcus aureus*	+	NA	+	PCR_validated
Sample028	*Staphylococcus aureus*	+	NA	+	PCR_validated
Sample046	*Staphylococcus aureus*	+	NA	+	PCR_validated
Sample077	*Staphylococcus aureus*	+	NA	+	PCR_validated
Sample088	*Staphylococcus aureus*	+	NA	+	PCR_validated
Sample050	*Staphylococcus aureus*	+	NA	+	PCR_validated
Sample022	*Staphylococcus aureus*	+	NA	+	PCR_validated
Sample060	*Stenotrophomonas maltophilia*	+	NA	–	tNGS_only
Sample098	*Stenotrophomonas maltophilia*	+	NA	–	tNGS_only
Sample153	*Stenotrophomonas maltophilia*	+	NA	+	PCR_validated
Sample194	*Stenotrophomonas maltophilia*	+	NA	+	PCR_validated
Sample115	*Stenotrophomonas maltophilia*	+	NA	+	PCR_validated
Sample110	*Stenotrophomonas maltophilia*	+	NA	+	PCR_validated
Sample106	*Stenotrophomonas maltophilia*	+	NA	+	PCR_validated
Sample103	*Stenotrophomonas maltophilia*	+	NA	+	PCR_validated
Sample105	*Stenotrophomonas maltophilia*	+	NA	+	PCR_validated
Sample109	*Stenotrophomonas maltophilia*	+	NA	+	PCR_validated
Sample116	*Stenotrophomonas maltophilia*	+	NA	+	PCR_validated
Sample210	*Stenotrophomonas maltophilia*	+	NA	+	PCR_validated
Sample114	*Stenotrophomonas maltophilia*	+	NA	+	PCR_validated
Sample101	*Stenotrophomonas maltophilia*	+	NA	+	PCR_validated
Sample090	*Stenotrophomonas maltophilia*	+	NA	+	PCR_validated
Sample151	*Stenotrophomonas maltophilia*	+	NA	+	PCR_validated

NA = not applicable.

Among these samples, the pathogens reported by tNGS and within the target range of RP40 included *Staphylococcus aureus, Stenotrophomonas maltophilia, Klebsiella pneumoniae, Acinetobacter baumannii, Moraxella catarrhalis, Pseudomonas aeruginosa, Haemophilus influenzae*, Human bocavirus. With the exception of *K. pneumoniae* and *A. baumannii*, the other pathogens exhibited a high degree of concordance in detection between tNGS and qPCR. For instance, out of 16 samples positive for *S. maltophilia* by tNGS, 14 were also detected by qPCR, and all samples positive for *M*. *catarrhalis* were detected by qPCR. In contrast, only 6 out of 11 samples positive for *K. pneumoniae* and 2 out of 8 samples positive for *A. baumannii* were detected by qPCR.

## Discussion

In recent years, several studies have highlighted the clinical utility of tNGS in the diagnosis of infectious diseases. A previous study revealed that tNGS presented a significantly higher positive detection rate and revealed higher pathogen variety and accuracy in bronchoalveolar lavage fluid samples (Li et al., 2022; Cui et al., 2024), indicating that tNGS has higher detection sensitivity. Compared with traditional culture methods, the tNGS method demonstrated excellent sensitivity and specificity in detecting periprosthetic joint infections and identifying microorganisms in culture-negative samples (Huang et al., 2023). Application research of tNGS technology in pediatric neurosurgery for central nervous system infection diagnosis showed that tNGS has high accuracy and sensitivity in determining the aetiology (Li et al., 2023). FA RP 2.1 has been clinically accepted with its core technology based on multiplex PCR, which shares similar principles with tNGS. Currently, there are no comparative studies examining tNGS in relation to FA RP 2.1 testing.

This study shows that among 190 nasopharyngeal swab samples, tNGS detected 34 pathogens, whereas FA RP 2.1 detected 12. tNGS covered a broader range of pathogens than FA RP 2.1 and was able to provide more detailed subtyping for Human respiratory syncytial virus and Human adenovirus, which FA RP 2.1 could not. The higher overall detection rate of tNGS compared with FA RP 2.1 may be contributed from the broader pathogen coverage. Regarding the 16 shared targeted spectrum, no significant difference between tNGS and FA RP 2.1 was observed on their positive rates, indicating that tNGS is as robust as FA RP 2.1 regarding the detection capability for these pathogens. However, it was observed that 26 samples had no pathogens detected by tNGS, suggesting that tNGS may need to expand its target pathogen coverage for upper respiratory tract infections.

Overall, tNGS presented high sensitivity and accuracy in reference to FA RP 2.1, but the instances of false negatives for Human respiratory syncytial virus and false positives for Influenza B virus, Human adenovirus, and Human rhinovirus indicated that the accuracy of tNGS in detecting these specific pathogens needs careful attention. The false negatives might be due to insufficient nucleic acid extraction or low primer amplification efficiency. On the other hand, false positives could result from low primer specificity. Therefore, optimizing these aspects of tNGS could potentially enhance its detection capability. Improving the nucleic acid extraction process could involve using more sophisticated extraction kits or protocols to ensure higher yield and purity of nucleic acids. Enhancing primer design to increase amplification efficiency and specificity could also mitigate both false positive and false negative results. Implementing these optimizations is likely to improve the accuracy and reliability of tNGS for detecting these and other pathogens.

A concern about this study is the discordance between tNGS and qPCR validation. Specifically, while tNGS detected *K. pneumoniae* in 11 samples and *A. baumannii* in 8 samples, subsequent qPCR validation using residual nucleic acid confirmed only 6/11 (54.5%) and 2/8 (25%) cases, respectively (**Supplementary Figure S3**). Several technical factors may explain this discrepancy: First, the prolonged sample storage may lead to nucleic acid degradation, particularly affecting samples with low bacterial loads - as evidenced by their low sequence reads in some samples. Second, inherent methodological differences between the assays, including variations in target gene selection and detection sensitivity, may contribute to the observed discordance. Notably, our quality control measures, including negative controls and bioinformatic reanalysis of target amplicons, effectively ruled out contamination or misclassification as potential confounding factors. While the current sample size (n=11 for *K. pneumoniae*; n=8 for *A. baumannii*) may limit definitive conclusions, these findings highlight the need for: (1) optimized sample handling protocols to minimize nucleic acid degradation, and (2) standardized comparative studies to better understand the performance characteristics of tNGS versus qPCR for bacterial detection in clinical samples.

Another limitation in this study is the inability of exploring the diagnosis value of tNGS. Due to institutional restrictions on patient data access and the absence of parallel treatment comparisons (each patient received only one therapeutic regimen), we could not directly evaluate whether detecting more pathogens via tNGS translates to superior clinical outcomes compared to FA RP 2.1. To address this gap, a future follow-up study should be incorporated: (i) standardized clinical outcome metrics (e.g., time-to-resolution, mortality), (ii) paired diagnostic comparisons (tNGS vs. FA RP 2.1 vs. culture), and (iii) rigorous assessment of tNGS’s impact on antimicrobial stewardship. This expanded design would clarify whether tNGS’s broader pathogen detection directly improves patient management.

The tNGS and FA RP 2.1 assays represent two distinct approaches to respiratory pathogen detection, each tailored to meet specific clinical needs (**Table 4**). The tNGS utilizes multiplex PCR combined with high-throughput sequencing, enabling the simultaneous screening of a broad target spectrum of 95 pathogens per assay, which optimizes it for fixed infection profiling. This method is optimized for batch processing, with the capacity to handle 96 samples in a single run, making it highly suitable for regular respiratory tract infection diagnostics. Besides, its turnaround time is approximately 12 hours from sample to report, and it offers a cost-effective solution with a per-test cost ranging from $69 to $110, especially when processing large batches to achieve marginal cost reduction.

**Table 4:** Comparison of key parameters between tNGS and FA RP 2.1.

Parameters	tNGS	FilmArray RP 2.1
Target Spectrum	95	22
Methodology	multiplex PCR & high throughput sequencing	fluorescent PCR (melting curve analysis)
Capacity	high throughput, 96 samples in a batch	lower throughput, 1–12 samples per run
Turnaround Time	12h (from sample to report)	full workflow autiomation, ~1h (from sample to report)
Recommended USE	simultaneously screens for 95 pathogen targets per assay, optimized for fixed infection profiling, recommended for regular respiratory tract infection	suitable for emergency department/point-of-care rapid diagnostics, recommended use for small-scale urgent testing
Cost	69~110$ per test	~420$ per test
Others	highly suitable for batch processing to achieve marginal cost reduction	requires no complex data analysis

In contrast, the FA RP 2.1 assay, with a target spectrum of 22 pathogens, employs fluorescent PCR and melting curve analysis, offering a lower throughput but with the advantage of full workflow automation. It can process 1 to 12 samples per run and deliver results in approximately 1 hour from sample to report, making it ideal for emergency department/point-of-care rapid diagnostics and small-scale urgent testing. Due to its relatively high per-test cost of around $420 and lower throughput, FA RP 2.1 is better suited for scenarios requiring immediate results and minimal hands-on time. Additionally, it requires no complex data analysis, further simplifying its use in urgent settings.

In summary, tNGS is well-suited for comprehensive and high-throughput screening in a laboratory setting with a focus on cost efficiency, while FA RP 2.1 excels in rapid, automated diagnostics for immediate clinical decision-making in emergency or point-of-care contexts.

Overall, the tNGS method can directly identify respiratory pathogens from specimens within 12 hours without the need for culture, and at a low cost. It shows a high degree of consistency with the FA RP 2.1 method, and exhibits high sensitivity and accuracy. This provides a new and feasible approach for diagnosing upper respiratory tract infections, aids in the formulation of early treatment plans, and holds the potential for widespread application in clinical practice.

## Data Availability

The data has been uploaded to the NCBI database. The original contributions presented in the study are publicly available. This data can be found here: PRJNA1293663.
