# Supplementary material for: Comparative performance evaluation of FilmArray RP 2.1 and targeted next-generation sequencing in upper respiratory tract infections
Source: Front Cell Infect Microbiol. 2025 Jul 24;15:1610445. doi: 10.3389/fcimb.2025.1610445 (PMC12329660; doi:10.3389/fcimb.2025.1610445)
Supplement: Supplementary file 1 [file DataSheet1.docx]

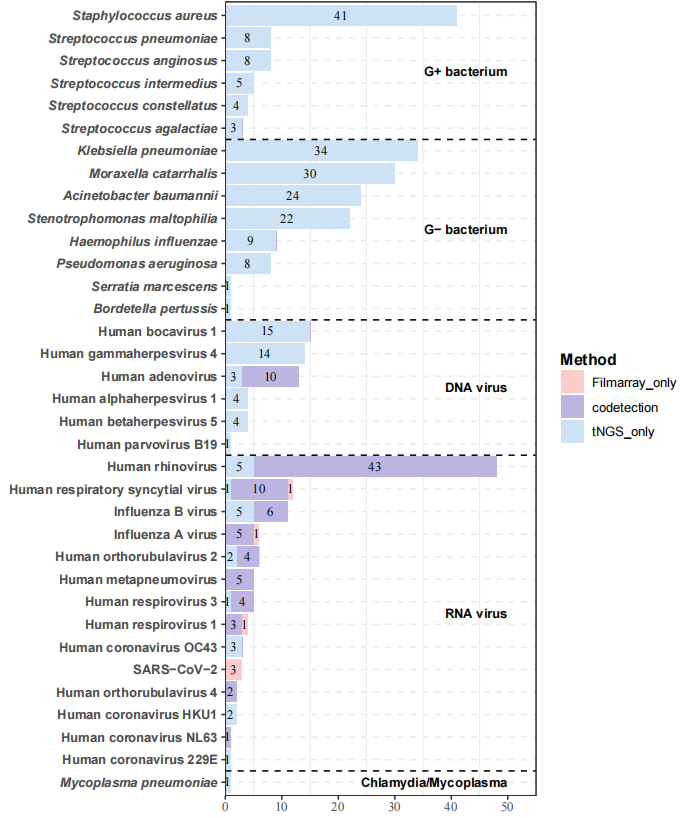


**Figure S1**: **Overlap of detection frequencies for each species (all targets) between FA RP 2.1 and tNGS.**


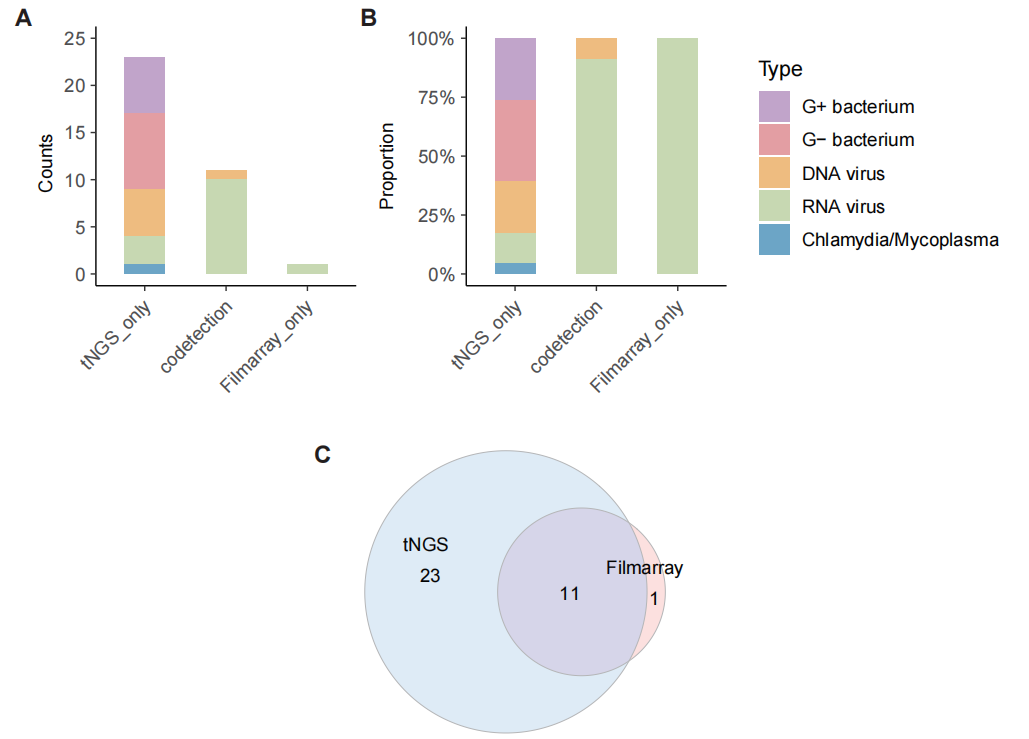


**Figure S2: Supplementary Analysis of Pathogen Detection between FA RP 2.1 and tNGS.**

Note**: A and B**: Detection frequencies / percentages of pathogen categories; **C**: Venn diagram showing the number of common or endemic reported pathogen types (including non-shared targets)


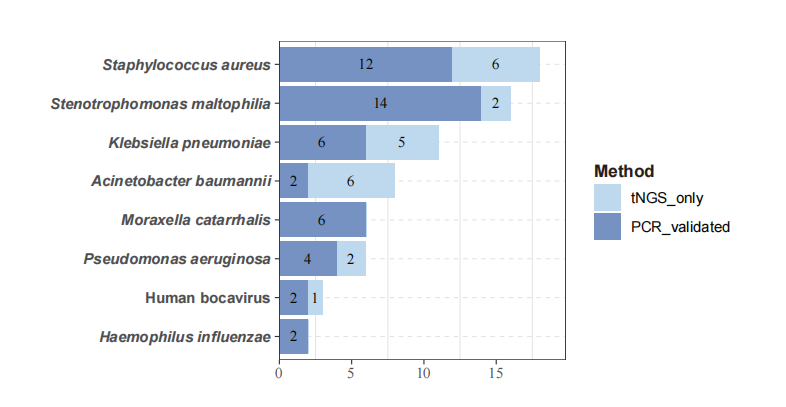


**Figure S3 Verification of selected pathogens reported by tNGS.**

Note**:** some of the pathogen species reported only by tNGS was validated using qPCR.
